# Antimicrobial Sensitivity Pattern of Salmonella Typhi: Emergence of Resistant Strains

**DOI:** 10.7759/cureus.11778

**Published:** 2020-11-29

**Authors:** Syed Asim Ali Shah, Muhammad Nadeem, Sarwar Ali Syed, Syeda Turab Fatima Abidi, Nasir Khan, Nazia Bano

**Affiliations:** 1 Medicine, Wah Medical College, Wah Cantt, PAK; 2 Medicine, Poonch Medical College, Rawalakot, PAK; 3 Pathology, Wah Medical College, Wah Cantt, PAK; 4 Biotechnology, International Islamic University, Islamabad, PAK

**Keywords:** antibiotic, extensively drug resistant (xdr), multidrug resistant (mdr), resistance, pakistan, salmonella typhi

## Abstract

Background

Typhoid fever is still an important public health problem in developing countries. Increasing resistance of Salmonella Typhi to antibiotics is alarming. New extensively drug-resistant strains of Salmonella reported first time in Pakistan, resistant not only to first-line drugs and ciprofloxacin but also resistant to ceftriaxone, had spread globally, including the USA. Due to this continuously changing pattern of antimicrobial resistance in typhoid fever due to Salmonella Typhi, there is a substantial need to study the resistance pattern of Salmonella Typhi frequently in different areas to detect the new resistant strains timely. The objective of this study was to evaluate the current trends in the resistance pattern of Salmonella Typhi in a tertiary care hospital in Northern Punjab.

Methods

This cross-sectional study was conducted at the Department of Medicine, Pakistan Ordnance Factories (POF) Hospital Wah Cant in collaboration with the Department of Pathology, from 1^st^ January 2019 to 30^th^ September 2019. Culture-positive patients of typhoid fever age more than 12 years, either male or female, were included in the study. The antimicrobial susceptibility of the isolates was determined by the disc diffusion method of Kirby Bauer on Mueller-Hinton agar using Clinical Laboratory Standards Institute (CLSI) guidelines. The antimicrobial agents tested were ampicillin (10 μg), chloramphenicol (30 μg), trimethoprim/sulfamethoxazole (1.25/23.75 μg), ciprofloxacin (5 μg), ceftriaxone (30μg), azithromycin (15μg), imipenem (10μg) and meropenem (10μg).

Results

A total of 81 culture-positive patients were included in the study. Out of these, 59% were male, and 41 % were female. Mean age was 23.8±19.1 years ranging from 12 to 91 years. Salmonella Typhi showed the highest sensitivity to imipenem 100% and azithromycin 95%; the lowest sensitivity was to ciprofloxacin 3.7%. Almost 50% of patients were resistant to ceftriaxone, and 48% were resistant to meropenem. The number of multidrug-resistant cases reported was 20%, whereas 47% of strains were extensively drug-resistant.

Conclusion

Resistance to antimicrobial agents is increasing in patients with typhoid fever due to Salmonella Typhi; especially the extensively drug-resistant strains of Salmonella Typhi are increasing rapidly. New emerging strains resistant to carbapenems found in our study are a big threat. Prescription of antibiotics according to culture and sensitivity for sufficient duration in patients of typhoid fever due to Salmonella Typhi is necessary to prevent the emergence of new resistant strains.

## Introduction

Typhoid fever is still an important public health problem in developing countries [1]. About 14.3 million cases were reported worldwide in 2017, with almost 136,000 deaths. Before the antibiotic era, mortality was more than 20%, but with the introduction of antibiotics, mortality reduced dramatically; in a study conducted in 2017, less than one percent mortality was reported [2]. Although antibiotics revolutionized the treatment of typhoid fever, the emergence of drug-resistant to first-line antibiotics (chloramphenicol, ampicillin, and trimethoprim/sulfamethoxazole) in the 1980s made treatment difficult and costly [3]. Fluoroquinolones were used as a drug of choice to treat multidrug-resistant (MDR) strains, but resistance to fluoroquinolones has increased rapidly due to the misuse of these antibiotics globally [4]. About 80-90% of patients were resistant to ciprofloxacin in many studies from Pakistan and other endemic areas, especially South Asia [5-7].

Increasing resistance of Salmonella Typhi is alarming; new extensively drug-resistant (XDR) strains reported first time in Pakistan, resistant not only to first-line drugs and ciprofloxacin but also resistant to ceftriaxone, had spread globally, including the USA [8]. Studies have shown that resistance to ceftriaxone is increasing globally [9, 10]. This rise in antibiotic resistance has increased morbidity, mortality, and treatment cost of typhoid fever [11, 12]. These XDR strains of Salmonella are sensitive to azithromycin, and costly carbapenems the mainstay of treatment for these patients [8].

Although no significant resistance to azithromycin and carbapenems is reported from Pakistan or other endemic areas, one study from Bangladesh showed many cases of azithromycin resistance strains of Salmonella Typhi [13]. This not only a great threat, but it also shows that there is a chance of the emergence of strains resistant to these antibiotics, which are the mainstay of treatment for XDR typhoid fever. Most of the studies also concluded that strains of Salmonella Typhi are usually resistant to multiple antibiotics, whereas resistance is not significant in Para Typhi strains [14]. So it is important to study the resistance pattern of Salmonella Typhi.

Due to this continuously changing pattern of antimicrobial resistance in typhoid fever due to Salmonella Typhi, there is a substantial need to study the resistance pattern of Salmonella Typhi frequently in different areas to detect the new resistant strains timely. Determination of local antibiotic sensitivity pattern is also important for empiric therapy. The objective of this study was to see the resistance pattern of Salmonella Typhi at a tertiary care hospital in Wah Cantt.

## Materials and methods

This cross-sectional study was conducted at the Department of Medicine, Pakistan Ordnance Factories (POF) Hospital Wah Cant in collaboration with the Department of Pathology, from 1st January 2019 to 30th September 2019. Culture-positive patients of typhoid fever age more than 12 years, either male or female, were included in the study by consecutive sampling. Patients with an obvious source of any other infection and having Salmonella Paratyphi strains on blood cultures were excluded. Informed consent was obtained from the patients or relatives wherever relevant. Ethical approval was taken from the hospital research ethics committee.

All blood samples were of venous origin and collected by standard sterile measures. Thus, in all selected cases, blood was cultured using the Bactec™ FX-40 system (Becton Dickinson, Franklin Lakes, USA). For each patient, 8-10mL of venous blood was drawn and inoculated in a special blood culture bottle (Bactec™ Plus Aerobic, anaerobic) containing 30 ml broth. Culture positive blood samples were subcultured on McConkey agar (Oxoid, Basingstoke, UK) and on blood agar (Oxoid, Basingstoke, UK) for 24 hours at 35 ± 2°C as per standard methods. API-20E (bioMerieux, Marcy l'Etoile, France), the analytical profile index system specific for differentiating between members of gram-negative Enterobacteriaceae, was utilized for biochemical testing. Serotyping was done by the group and type-specific antisera (Bio-Rad, Hercules, USA).

The antimicrobial susceptibility of the isolates was determined by the disc diffusion method of Kirby Bauer on Mueller-Hinton agar using Clinical Laboratory Standards Institute (CLSI) guidelines. The antimicrobial agents tested were ampicillin (10 μg), chloramphenicol (30 μg), trimethoprim/sulfamethoxazole (1.25/23.75 μg), ciprofloxacin (5 μg), ceftriaxone (30μg), and azithromycin (15μg). Imipenem (10μg) and meropenem (10μg) were tested when extended sensitivity was required. However, azithromycin interpretation was based on the disc manufacturer’s recommendations.

MDR was defined as Salmonella strains resistant to ampicillin, trimethoprim/sulfamethoxazole, and chloramphenicol, whereas XDR was defined as strains resistant to ampicillin, trimethoprim/sulfamethoxazole, chloramphenicol, ciprofloxacin, and ceftriaxone [15].

Data was entered, and statistical analysis was done in SPSS version 20 (IBM Inc., Armonk, USA). Frequencies and percentages were calculated.

## Results

A total of 81 culture-positive patients were included in the study. Out of these, 59% (n=48) were male and 41 % (n=33) were female. The mean age was 23.8±19.1 years ranging from 12 to 91 years. Majority of the patients presented with fever alone, i.e., 96% (n=78), 4% (n=3) presented with fever and gastrointestinal symptoms. About 58% (n=47) patients were admitted to the medical ward, whereas 42% (n=34) were treated in the outpatient department (OPD). All patients recovered completely; no death was reported during the study period. Salmonella Typhi showed the highest sensitivity to imipenem 100 (n=39) and azithromycin 95% (n=77), lowest sensitivity was to ciprofloxacin 3.7% (n=3). Number of MDR cases reported were 20% (n=16), whereas 47 % (n=38) cases were XDR. Sensitivity and resistance pattern to common antimicrobial agents is shown in Table 1, whereas sensitivity and resistance pattern to carbapenems is shown in Table 2.

**Table 1:** Sensitivity and resistance pattern of Salmonella Typhi to common antimicrobial agents

Antibiotic	Sensitive	Partially sensitive	Resistant
Ampicillin	14.8%		85.2%
Azithromycin	95%		5%
Ceftriaxone	49.4%	1.2%	49.4%
Cotrimoxazole	33.3%		66.7%
Ciprofloxacin	3.7%	1.2%	95%
Chloramphenicol	26%		74%

**Table 2:** Sensitivity and resistance pattern of Salmonella Typhi to imipenem and meropenem

Antibiotic	Sensitive	Partially sensitive	Resistant
Imipenem	100%	0%	0%
Meropenem	41%	11.3%	47.7%

## Discussion

The incidence of XDR cases reported in our study was very high. The incidence of MDR was 20%, whereas the incidence of XDR was 47% - this is an alarming situation. Almost 95% of cases were resistant to ciprofloxacin, commonly used as a first-line drug against Salmonella Typhi in Pakistan. Resistance was also very high against ceftriaxone and second-line injectable antibiotic (meropenem) against Salmonella Typhi. Azithromycin and Imipenem were the only drugs with a sensitivity of more than 90%.

MDR strains of Salmonella Typhi are common worldwide, and many outbreaks of MDR are reported from South Asia, South East Asia, Africa, and China in the recent past [16-18]. Globally prevalence of MDR typhoid fever varies from 10-80% in endemic areas [19-21]. This is comparable to our findings. Increased incidence of MDR typhoid fever was especially reported from different areas of Pakistan in recent years; MDR Salmonella strain was found in 64-66% of patients in a study conducted in Pakistan [5], our study showed MDR strain in 20% of patients. This difference may be due to increased sensitivity to cotrimoxazole and chloramphenicol in some areas in recent years as the use of these antibiotics has reduced. However, a recent study showed only 2.6% of MDR typhoid cases in children [22]. This inconsistency with our results as well as other studies from Pakistan may be due to an outbreak of low resistant strain in a specific area of that particular hospital; otherwise, most of the studies from Pakistan have almost the same results as in our study [6].

Our study showed that 47% of patients had XDR strains of Salmonella Typhi. The emergence of XDR typhoid is a global threat. Many local and international studies support our findings. Pakistan is among those countries where outbreaks of XDR typhoid have been reported recently [23]. More than five thousand cases of XDR typhoid were reported during these outbreaks [24]. Another study showed that XDR strains are present all over Pakistan [25]. XDR strains of Salmonella Typhi are common in Asia and areas of Africa [4]. Our results are comparable with all these findings. The emergence of the XDR strain in South Asia and especially in Pakistan may be due to the injudicious use of antibiotics. Antibiotics are available in Pakistan over the counter without any prescription. Antibiotics are also prescribed injudiciously by doctors in Pakistan and other parts of South Asia [26]. Judicious use of antibiotics is necessary to stop the emergence of such superbugs.

Resistance to ciprofloxacin was very high in our study - 95% of patients were resistant to ciprofloxacin. Other studies from Pakistan also had the same findings - 91.7% of patients with typhoid fever were resistant to quinolones in a study conducted by Qamar et al. [5]. Fluoroquinolones remained the treatment of choice for enteric fever for more than two decades, but high resistance to these drugs has been reported now from different areas of the world, especially from South Asia [4]. This also supports our findings.

Almost 50% of patients were found resistant to ceftriaxone in our study; studies from different parts of the world also show that ceftriaxone resistance is increasing [9, 10]. However, some studies from Pakistan have shown less than one percent resistance to ceftriaxone [6]. This contrast with our findings may be due to differences in the catchment area; another reason is that the study was conducted five years ago, and resistance has probably increased during these five years due to the increasing use of ceftriaxone as a first-line drug against enteric fever.

After the emergence of XDR strains of Salmonella Typhi, physicians are increasingly using carbapenems [4]. The real threat found in our study was high resistance to carbapenems; 48% were found resistant to meropenem. The XDR strains earlier reported from Pakistan were susceptible to carbapenems and azithromycin, which were the main treatment options for these cases. Almost 90% of patients were sensitive to imipenem and meropenem in a study from Pakistan [23]. Regarding imipenem, findings in this study are comparable with our findings, but the emergence of meropenem resistant strain of Salmonella Typhi is an alarming public health concern as there are fewer antibiotics for the treatment of XDR typhoid. It is high time to take some important steps to deal with this public health problem before it is too late. Vaccination against typhoid fever, good hygiene, stopping the unnecessary use of antibiotics, testing blood culture of patients with suspected Typhoid fever before starting antibiotic and long enough (10-14 days or afebrile more than five days, whichever is longer) use of antibiotics for typhoid fever are the few steps that should be taken immediately.

Our study had a few limitations. First, it was a single-center study. Secondly, we did not look for factors responsible for increased resistance, like recent use of antibiotics, source of water supply, eating habits, etc. In future multicenter studies to confirm our findings of high resistance to carbapenems and exploring the factors for increasing resistance are suggested.

## Conclusions

Resistance to antimicrobial agents is increasing in patients with typhoid fever due to Salmonella Typhi; especially the extensively drug-resistant strains of Salmonella Typhi are increasing rapidly. New emerging strains resistant to carbapenems found in our study are a big threat. Prescription of antibiotics according to culture and sensitivity for sufficient duration in patients of typhoid fever due to Salmonella Typhi is necessary to prevent the emergence of new resistant strains.
